# Dynamic Microstructure Assembly Driven by *Lysinibacillus* sp. LF-N1 and *Penicillium oxalicum* DH-1 Inoculants Corresponds to Composting Performance

**DOI:** 10.3390/microorganisms10040709

**Published:** 2022-03-25

**Authors:** Haiyan Duan, Cong Fu, Guilin Du, Shiqiu Xie, Min Liu, Baoguo Zhang, Jiping Shi, Junsong Sun

**Affiliations:** 1 Laboratory of Biorefinery, Shanghai Advanced Research Institute, Chinese Academy of Sciences, No. 99 Haike Road, Shanghai 201210, China; duanhaiyan@sari.ac.cn (H.D.); fuc@sari.ac.cn (C.F.); dugl1@shanghaitech.edu.cn (G.D.); xieshiqiu2019@sari.ac.cn (S.X.); liumin1@shanghaitech.edu.cn (M.L.); zhangbg@sari.ac.cn (B.Z.); 2 School of Life Science and Technology, Shanghai Tech University, Shanghai 201210, China; 3 University of Chinese Academy of Sciences, Beijing 100049, China

**Keywords:** compost, *Lysinibacillus*, *Penicillium*, microbial community, functional prediction

## Abstract

The effects of *Lysinibacillus* sp. LF-N1 and *Penicillium oxalicum* DH-1 inoculants (LFPO group) on compost succession and the microbial dynamic structure of co-composting wheat straw and cow manure composting were investigated. The inoculants contributed to longer thermophilic stages, higher temperatures (62.8 °C) and lower microbial diversity in the LFPO treatment compared to the control group (CK). Moreover, LFPO inoculation increased the germination index and accelerated organic matter and lignocellulose degradation in the compost. Microbial analysis confirmed that the inoculants effectively altered the microbial communities. The predominant biomarkers for bacteria and fungi in inoculated compost were members of *Lysinibacillus* and *Penicillium*, respectively. Functional prediction showed greater lignocellulose degradation and less pathogen accumulation in the LFPO group. The cooccurrence network analysis showed that the network structure in LFPO compost was greatly simplified compared to that in CK. Bacterial cluster A was dominated by *Lysinibacillus*, and fungal cluster B was represented by *Penicillium*, which were significantly correlated with temperature and lignocellulose degradation, respectively (*p* < 0.05). These results demonstrated that the LF-N1 and DH-1 inoculants drove the bacterial and fungal assemblies to induce physicochemical property changes during cocomposting.

## 1. Introduction

The number of 270 million dairy cows in the world makes for a huge share of cow manure, which currently accounts for more than half of the total manure and is estimated to account for around 75% of the total fertilizer production in the EU by 2030. China’s annual production of animal manure can reach approximately 3.8 billion tons [1]. In the production of rice, the amount of straw waste can reach approximately 200 million tons per year [2]. The environmental protection facilities of large-scale intensive farms are not sufficient to deal with these manures in a timely and centralized manner. Generally, these manure and straw wastes are discarded via open dumping, landfilling or burning, which could not only cause serious pollution to the surrounding environment, but also harm the health of dairy cows, even damaging the economics of the farm. In order to reduce the above risks to the environment and realize the recyclable utilization of these waste resources, it is necessary to find sustainable and cost-effective waste recycling systems as much as possible.

Composting is defined as a microbially involved, aerobic process accompanied by physiological and biochemical reactions, which involves the thermophilic stage and the hydrolysis of organic matter (OM) into humic-like substances that are stable and non-toxic. For improving the degradation efficiency of organic substances and the humification degree, various microorganisms have been inoculated during composting [3,4]. Several studies suggested that the inoculation of efficient microbes (EM) into different organic waste composts can effectively improve the waste degradation rate. These inoculated microorganisms can be isolated from microbial communities based on specific degradation functions, or by isolation and screening from culture mixtures such as soil, manure and seawater [5,6]. Certain composting investigations indicated that bio-agents in a compost mixture effectively alter various characteristics of the composting process, such as increased temperature [7], changed pH value and the C/N ratio [5,6]. *Streptomyces* inoculation significantly preserves the nutrients in the compost, such as N, P, K [8]. Inoculation with psychrophilic and thermophilic microbial inoculants can improve the cow manure composting efficiency [5].

Lignocellulose, as the main component of agricultural waste, which consists of cellulose, hemicellulose and lignin, is a refractory carbon-containing compound in agricultural waste, which accounts for the slow degradation of compost and thereby limits the formation of humus [9,10]. Some microorganisms have been used as effective compost inoculants because of their ability to secrete cellulase to hydrolyze cellulose, and then degrade organic agricultural waste [11,12]. Several trial schemes indicated that microbial inoculation results in the secretion of cellulases and the acceleration of the biodegradation of cellulose during composting [10,11]. Some *Penicillium* species produce cellulases and hemicellulases [13,14]. Compost inoculated with *Penicillium expansum* exhibited greater lignocellulose degradation that proceeded 50% faster compared with the absence of inoculation [15]. Some species of *Lysinibacillus* are reported to be cellulose- and lignin-decomposing bacteria [16,17,18]. However, there are few cases of the simultaneous inoculation of bacteria and fungi into compost. It is unclear how bacterial and fungal complex inoculants affect the chemical structure and microstructure. Moreover, the driving effects of the selected functional bacterial–fungal mixed inoculum on bacterial and fungal community structural dynamics are still unknown.

In this study, a consortium of enzymatic and acid odor-degrading multifunctional strains were isolated from cow manure and inoculated to the compost. By monitoring the composting process of the control group (CK) and the inoculated group (LFPO), the differences between the two groups were compared. This research aimed to (a) investigate the influence of LFPO on the composting performance, such as physicochemical properties and the degradation of lignocellulose; (b) explore the microbial community dynamics during composting; (c) discuss the interaction among physicochemical properties and bacterial and fungal communities.

## 2. Materials and Methods

### 2.1. Microbial Inoculant Preparation

Highly efficient acid-degrading strains, namely *Lysinibacillus* sp. LF-N1 (CCTCC No: M 2017289) and *Penicillium oxalicum* DH-1 (CCTCC No: M 2018201), were previously isolated in our laboratory from cow manure. The *Lysinibacillus* sp. LF-N1 strain was cultivated in LB medium at 37 °C and 200 rpm. The suspension (1 × 10^8^ CFUs mL^−1^) was used as an inoculant. *Penicillium oxalicum* DH-1 slants were stored on PDA medium at 4 °C. The strains were inoculated into 250 mL Erlenmeyer flasks (containing 100 mL PDA medium). Sterile glass beads (8 mm) were placed in each Erlenmeyer flask and incubated for 5 days on a rotary shaker at 150 rpm at 30 °C. Cultures were collected by filtration and washed three times with 90 mL of 0.12 M NaH_2_PO_4_-Na_2_HPO_4_ buffer to reach 1 × 10^8^ CFUs mL^−1^. After that, these mycelial preparations (<3 mm) were used as inoculants. For inoculation, these two candidate strains were mixed (1:1, *w*/*w*). The inoculants were added at a ratio of 2% dry weight [7]. The control group (CK) was treated with an equivalent amount of inactivated LF-N1 and DH-1 fermentation broth sterilized by an autoclave (121 °C).

### 2.2. Composting and Sampling

Cow manure was obtained from Ji Xiang Farm, Co., Ltd., Fujian, China, and was collected after solid–liquid separation. Wheat straw was cut into 20–30 mm pieces for use as a composting additive. The characteristics of the cow manure are shown in Table 1. The C:N ratio and water content of the original compost were set at 25:1 and 60% [8,12]. Composting experiments were carried out in laboratory-scale aerobic composting systems [19]. The total weight of each pile was approximately 10 kg, and each compost treatment was carried out in a special cylindrical compost reactor (5 L) (Figure S1). All of the groups were replicated three times. The composting lasted for 40 days, and the composts were turned over on days 0, 7, 15, 20, 25 and 40 to enhance aeration.

### 2.3. Physicochemical Analysis

The temperature was recorded at the top, middle and bottom of the compost. Samples were collected in the initial phase (day 0), mesophilic phase (day 3), thermophilic phase (day 7), cooling phase (day 20) and maturation phase (day 40) according to the composting process [20], and were stored at −20 °C before analysis. Samples were collected after drying at 105 °C for over 24 h until reaching a constant weight; then, we ground and sieved the samples (40 mesh sieve), and determined total organic carbon (TOC) using potassium dichromate spectrophotometry according to the Chinese national standard (HJ 615-2011). Total nitrogen (TN) was measured by the Kjeldahl method [12,21]. The C/N ratio was calculated using TOC/TN. OM was measured by placing samples in a muffle furnace at 550 °C for 6 h [4]. The content of lignocellulose, containing cellulose, hemicellulose and lignin, was determined with an ANKOM220 Fiber Analyzer using the method described by Van Soest et al. [22]; the hemicellulose content was estimated as the difference between neutral-detergent fiber (NDF) and acid-detergent fiber (ADF); the cellulose was estimated as the difference between ADF and acid-detergent lignin (ADL); and the lignin was estimated as the difference between ADL and ash content.

The fresh samples and distilled water were mixed at a ratio of 1:10 (mass concentration), and then the pH was measured by pH meter PB-10 (Sartorius, Göttingen, Germany). The above mixed solution was vibrated and centrifuged (4000 g·min^−1^) to obtain an extract, and the extract was used to infiltrate the cabbage seeds, and cultivated at 25 °C for 48 h. The germination index (GI) was determined by combining measurements of germination and root elongation of cabbage seeds, followed by procedures as described [21],

$$\mathrm{GI}\;\left(\%\right)=\frac{\mathrm{Seed}\;\mathrm{germination}\;\mathrm{rate}\;\times \;\mathrm{length}\;\mathrm{of}\;\mathrm{treatment}\;\left(\mathrm{mm}\right)}{\mathrm{Seed}\;\mathrm{germination}\;\mathrm{rate}\;\times \;\mathrm{length}\;\mathrm{of}\;\mathrm{control}\;\left(\mathrm{mm}\right)}\times 100$$

### 2.4. DNA Extraction

Total DNA of the microbial community was extracted using an E.Z.N.A.^®^soil DNA Kit (Omega Bio-Tek, Norcross, GA, USA). The DNA purity and concentration were measured using a NanoDrop 2000 UV–vis spectrophotometer (Thermo Scientific, Wilmington, NC, USA). Samples for DNA analysis were stored at −80 °C until required. PCR amplification was performed as described previously [23]. The V3–V4 regions of 16S rRNA were amplified with the barcoded fusion primers 338F (ACTCCTACGGGAGGCAGCAG) and 806R (GGACTACHVGGGTWTCTAAT). The ITS region from ITS rRNA was amplified using ITS1F (CTTGGTCATTTAGAGGAAGTAA) and ITS2R (CTTGGTCATTTAGAGGAAGTAA). PCR products were sent to Majorbio Biopharm Technology Co., Ltd. (Shanghai, China) for further high-throughput sequencing, as described previously [24].

### 2.5. Bioinformatics Analyses

Sequences obtained by high-throughput sequencing were quality-controlled by QIIME1.9.1 (v. 1.9.1 http://qiime.org/, accessed on 23 February 2022), and low-quality sequences were removed. The quality-filtered sequences were clustered into operational taxonomic units (OTUs) with a 97% similarity threshold using UPARSE (v. 7.1 http://drive5.com/uparse/, accessed on 13 December 2021). The taxonomy of sequences was annotated by alignment with Silva 138 (https://www.arb-silva.de/, accessed on 11 January 2022). The diversity index and richness index were calculated using the vegan package in the R software (v.3.4.1). The significantly different taxa in different groups were identified using the Wilcoxon rank-sum test (*p* < 0.05). PICRUSt (http://picrust.github.io/picrust/, accessed on 13 December 2021) was used to infer metabolic functions of bacterial communities in compost samples, and we used KEGG to analyze bacterial metabolic pathways during composting. The bacterial phenotypes were manually annotated using the DacDive database (the Bacterial Diversity Metadatabase; https://bacdive.dsmz.de/, accessed on 13 December 2021) [25]. Fungal library FUNGuild (http://www.stbates.org/guilds/app.php, accessed on 13 December 2021) was used for fungal function prediction [26]. Construction of a neighbor-joining phylogenetic tree was performed using MEGA 6.0. The molecular ecological network of bacteria and fungi during composting was constructed using Gephi software (v. 0.9.2) to analyze the interaction network of microorganisms during composting. One-way analysis of variance (Tukey HSD) was used to determine the statistical differences in physical and chemical factors in the composting process, and the significance level of the differences was 0.05.

## 3. Results

### 3.1. Variations in Physicochemical Properties

**Figure 1:**
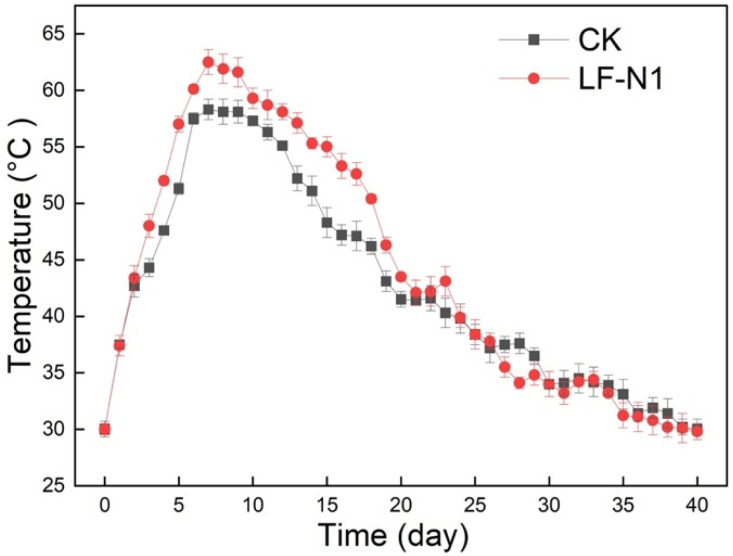
The temperature profile throughout composting.

Temperature is an important environmental factor that affects the metabolic activities of microorganisms and thus influences composting efficiency as compost proceeds [7]. Figure 1 shows the evolution of temperature in the CK and LFPO treatments. During the mesophilic phase of composting (<50 °C), the composting temperature showed continuous elevation, mainly because heat-tolerant microorganisms generated heat, ascribed to the degradation of organic fractions, such as hydrolyzable carbohydrates, fats, proteins, etc. [19]. Subsequently, the two treatments reached temperatures higher than 50 °C on the 5th (LFPO) and 6th (CK) days, entering the thermophilic phase. The temperature of the compost spontaneously rose to a maximum of 62.8 °C (day 7) in LFPO and 58.3 °C (day 8) in CK. The high-temperature duration (thermophilic phase) of LFPO compost (15 days) was longer than that of the CK group (9 days) due to the inoculation of the two strains, LF-N1 and DH-1, in this experiment. Unlike shortening the heating phase of composting by other microbial inoculants [3], the present study indicated that the LF-N1 and DH-1 inoculants contributed to a higher temperature and longer thermophilic phase. Similar results are described in [8]. This result showed that the LF-N1 and DH-1 inoculants increased the compost temperature and maintained the high-temperature phase. Several trial schemes found that several commercial microbial inoculants slowed down the composting process, probably due to the excessive heat deactivating the microbial agent and then hastening composting [27]. Compared with the inoculation of lactic acid bacterium during composting, in which the temperature began to increase after day 8 and reached 60 °C on day 10 [6], LF-N1 and DH-1 inoculants advance compost heating. Other studies have also reported the insignificant effect of commercial microbial inoculants when compared to the control treatment; this could be because of the suppression of inoculants at higher temperatures during composting [27]. After the thermophilic phase, the temperature gradually decreased to less than 50 °C, entering the cool and mature phase, and was stable at approximately 30 °C. Hence, the LF-N1 and DH-1 inoculants showed advantages in improving the quality of compost by extending the thermophilic period.

The physical and chemical properties in the compost matrix are shown in Table 2. The TOC and the C/N ratio values significantly decreased as composting progressed, while the TN content and GI value significantly increased in all groups. The pH was elevated amidst composting and then decreased, and both CK (8.04) and LFPO (7.87) finally stabilized at suitable pH values (7.0–8.5) for compost maturity [28]. The inoculated treatment resulted in a lower pH value throughout the whole composting process (*p* < 0.05). The TOC content rapidly decreased from 374.6 to 282.3 g·kg^−1^ in CK, and the final TOC content in LFPO (269.6 g·kg^−1^) was significantly lower than that in CK (*p* < 0.05). The greater reduction in TOC indicates the continuous biodegradation of carbon-containing substances and the formation of CO_2_ by more active microorganisms in incubated compost. There was no significant difference between the two treatments in the ability to preserve *N* (16.1 g·kg^−1^ in CK vs. 16.8 g·kg^−1^ in LFPO, *p* > 0.05). The “concentration effect”, stating that the organic fraction is lost in the form of gases such as H_2_O and CO_2_, contributed to higher N content per unit part of compost mass; similar results were reported for inoculations with a psychrotrophic–thermophilic complex microbial agent during manure–rice straw composting [5,8]. The C/N ratio declined as composting proceeded; furthermore, we found a lower rate of reduction in the C/N ratio in LFPO (*p* < 0.05). The final C/N ratio of 16.8 of the LFPO was observed, which was a good result (<17) for a favorable compost product with high quality [29]. The germination index is a vital parameter to induce nontoxicity and maturity of compost products (>80%) [30]. The GI value of the LFPO group exceeded 80% on the 7th day, and it gradually increased in the later period. The GI value in LFPO was significantly higher than that in CK in process time (*p* < 0.05). The final GI values in both the CK (125.4%) and LFPO (177.1%) groups met the standard for compost maturity. No significant variations in pH and C/N ratio were found in the LFPO (*p* < 0.05) and CK (*p* < 0.001) treatments, and significant variations in pH, TOC, the C/N ratio and GI (*p* < 0.001) with composting time were observed. The above suggests that multiple parameters in the composting process, such as temperature, pH and the C/N ratio, determined the fact that microbial combinations with LFPO accelerated the maturity and safety of the compost products.

### 3.2. Degradation of Organic Matter and Lignocellulose

The degradation of lignocellulose is shown in Table 3. The OM content in the LFPO group declined throughout the composting, but OM in CK decreased early in the composting process until there was no significant difference after day 20 (*p* > 0.05). The LFPO treatment presented greater OM degradation than CK during the whole composting process (*p* < 0.05). A similar result was reported wherein thermophilic microbial consortium inoculation enhanced the OM degradation and then could improve humification during composting [7]. Regarding cellulose and hemicellulose degradation, compared to the CK group, the cellulose and hemicellulose content in the LFPO group decreased rapidly in the thermophilic phase and stabilized on day 20 since there was no significant change between days 20 and 40 with time (*p* > 0.05), while no significant difference was observed during the thermophilic phase in CK but the values decreased significantly upon entering the late phases. There were significant treatment differences in the degradation of cellulose and hemicellulose (*p* < 0.001). Within the cooling phase, the declined rate of cellulose biodegradation was 65.7% (LFPO) and 46.7% (CK), while hemicellulose degradation was 80.6% and 66.8%, respectively. Lignin is a complex organic polymer that is usually interconnected with cellulose and hemicellulose to form a ligno-carbohydrate complex that is resistant to biodegradation [31]. With respect to lignin degradation in the two treatments, there were no significant differences in the first 3 days (*p* > 0.05), but lignin degradation decreased rapidly after 7 days along with composting time, indicating that a series of microorganisms related to the degradation of lignin may have begun action in the later stage of composting. Wang et al. reported that microorganisms require temperatures even higher than 60 °C to degrade cellulose and hemicellulose, and that lignin degradation mainly occurs at 50 °C, which could explain the trends in the degradation of lignocellulose during co-composting [12,32]. Overall, there was a significant difference between the LFPO and CK treatments (*p* < 0.05) in the degradation of OM, cellulose, hemicellulose and lignin, and there was a significant difference with composting time (*p* < 0.001), indicating that LFPO incubation composting significantly accelerated the degradation of OM and lignocellulose, which was consistent with the results presented by Xu et al., where lignocellulolytic inoculants including *Phanerochaete chrysosporium* and *Streptomyces griseorubens* accelerated the degradation of cellulose and hemicellulose in the initial stage of composting and therefore enhanced the whole composting process eventually [10].

### 3.3. Similarity and Diversity of the Microbial Community

Table 4 shows the abundance and diversity index of microbial sequencing, which can fully represent the real situation of bacterial and fungal community structures during composting for further bioinformatics analysis. The coverage index of all samples was above 0.99. Chao1 and Ace are indexes reflecting community richness, while the Shannon and Simpson indexes reflect community diversity. The larger the Chao1 index, the higher the richness of the microbial community, while the larger the Simpson index, the lower the microbial diversity. The bacterial Shannon index first markedly increased followed by a decrease, and the Simpson index significantly increased and then declined in LFPO compared with CK (*p* < 0.05), indicating that the species richness of the inoculated group (LFPO) decreased compared with CK during the microbial active period; furthermore, there was no significant difference between the Shannon and Simpson indexes of fungi or the Chao1 and Ace indexes of bacteria (*p* > 0.05).

### 3.4. Microbial Dynamics Analysis

#### 3.4.1. Bacterial Community Succession

**Figure 2:**
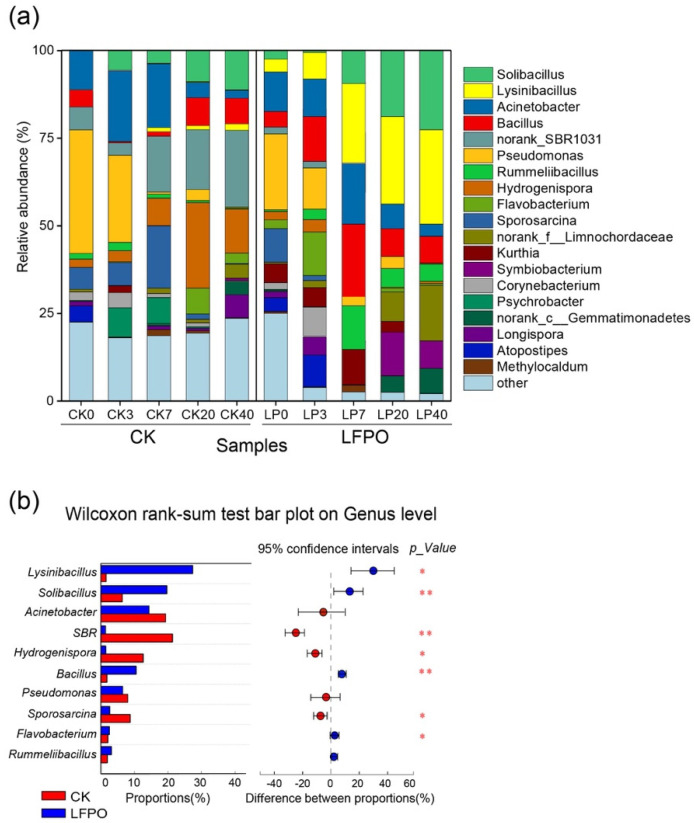
The bacterial community structures in cow manure and wheat straw compost with and without inoculation. (**a**) The bacterial taxa at the genus level; taxa with <1% reads were combined as “others”. (**b**) Wilcoxon rank-sum analysis of significant bacterial genera between silages without and with inoculation; **, and * represent *p* < 0.01, and *p* < 0.05, respectively.

Composting time and LFPO inoculation significantly reshaped the bacterial community structure during composting (*p* < 0.05, Figure 2a). With respect to the raw materials, Proteobacteria was the most dominant phylum (62.9%), followed by Firmicutes (28.1%) and Actinobacteria (7.8%) (Figure S2a). The most abundant genera included *Pseudomonas* (24.7–34.5%), *Acinetobacter* (8.4–12.2%), norank_o__SBR1031 (6.7%) and *Sporosarcina* (6.8–11.3) (Figure 2a). The 40-day composting process effectively enriched Firmicutes, which replaced Proteobacteria as the most abundant phylum in the present study, which was different from the results obtained by Chi et al., who found that when compost with *Streptomyces griseorubens JSD-1* inoculation was used, the RA of Firmicutes decreased, while the RA of Chloroflexi increased and Chloroflexi played a significant role in swine manure and rice straw co-composting [8]. In CK, the undesired norank_o__SBR1031 was observed with the highest RA of 35.8%, together with *Hydrogenispora* (23.7%), and the RA of *Lysinibacillus* was only 2.3% at the end of the composing. Under LFPO inoculation, in the thermophilic phase, *Bacillus* and *Lysinibacillus* significantly increased in the LFPO and were significantly higher than those in the CK (*p* < 0.01). The RAs of *Lysinibacillus*, *Bacillus* and *Acinetobacter* increased sharply from the 7th day, and these were the dominant taxa until the end of composting. Notably, in the LFPO group, *Lysinibacillus fusiformis* was the most abundant species during the mature phase of composting (Figure S3a). As Figure 2b shows, *Lysinibacillus* (25.8%), *Bacillus* (24.5%) and *Solibacillus* (11.1%) were more enriched in the LFPO group (*p* < 0.05) during the thermophilic phase than in the CK group (*p* < 0.01). *Acinetobacter* existed in both groups (14.8% in LFPO and 18.7% in CK). Norank_o__SBR1031 and *Sporosarcina* significantly increased in CK and were significantly higher than those in LFPO (*p* < 0.05). The RA of *Pseudomonas*, which was similar between the two groups, gradually decreased during composting as the temperature increased, which may be due to its intolerance of high temperatures. Other genera (RA > 0.01) accounted for more than 20% in the CK, less than 3% in the LFPO, indicating that LFPO incubation increased the aggregation of dominant bacteria.

#### 3.4.2. Fungal Community Succession

**Figure 3:**
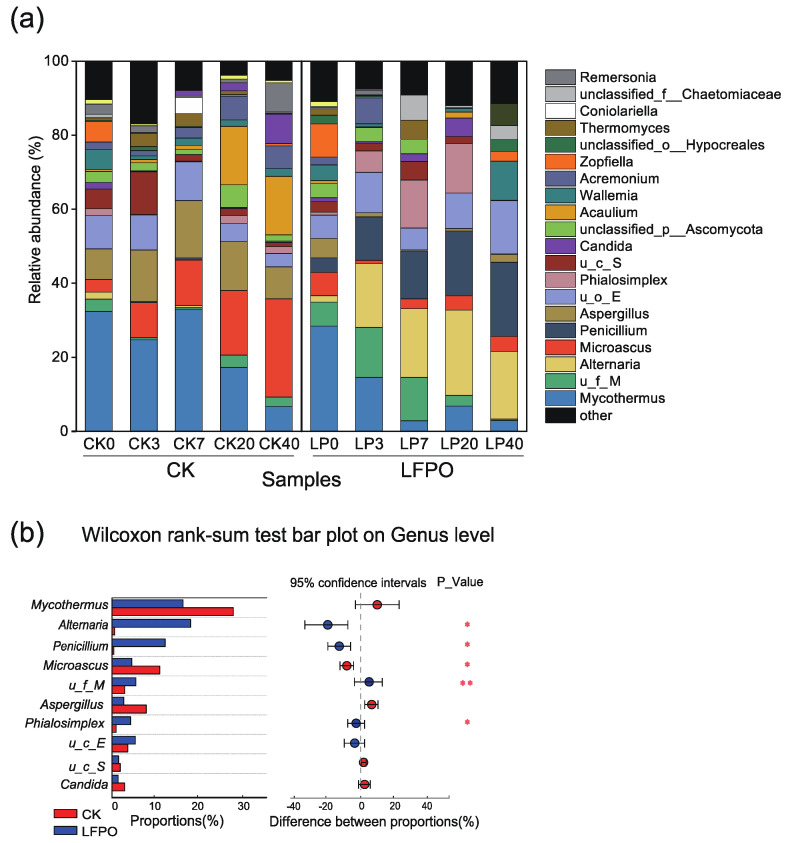
The fungal structures during composting. (**a**) The relative abundance of fungal taxa at the genus level; taxa with <1% reads were combined as “others”. (**b**) Wilcoxon rank-sum analysis of significant fungal genera between compost without and with inoculation; **, and * represent *p* < 0.01, and *p* < 0.05, respectively.

The composting time and LFPO inoculation also significantly reshaped the fungal community (Figure 3 and Figure S2b, *p* < 0.05). The original fungal genera in the raw materials were mainly *Mycothermus* (33.4%, Ascomycota), *Aspergillus* (8.4%, Ascomycota) and *Wallemia* (7.4%, Basidiomycota). Entering the late stage of composting, the most abundant taxon changed to *Alternaria* (22.4%), followed by *Penicillium* (21.6%), unclassified_o__Eurotiales (13.1%) and *Wallemia* (10.7%) in the LFPO group. *Alternaria* (19.8%), *Penicillium* (14.3%), unclassified_f__Microascaceae (17.7%) and *Phialosimplex* (12.1%) were more enriched in the LFPO group (*p* < 0.05), while *Mycothermus* (32%), *Aspergillus* (17.8%) and *Microascus* (10.3%) were more enriched in the CK group (*p* < 0.05) during composting (*p* < 0.05) (Figure 3b). Unclassified_o__Eurotiales was found in both groups during composting, indicating that unclassified_o__Eurotiales probably had the ability to adapt to more types of environments. In the thermophilic phase, *Alternaria* and *Penicillium* increased in the LFPO compost and were significantly more abundant than those in the CK (*p* < 0.05). It is worth noting that inoculation with LF-N1 and DH-1 significantly enriched *Alternaria* by 93.5% compared with the uninoculated group in this study. In addition, in the LFPO group, *Penicillium oxalicum* was the most abundant species during the mature phase of composting (Figure S3a).

### 3.5. Effects on the Microbial Dynamicd and Function Prediction

#### 3.5.1. Effects on the Phenotype of Bacterial Communities

**Figure 4:**
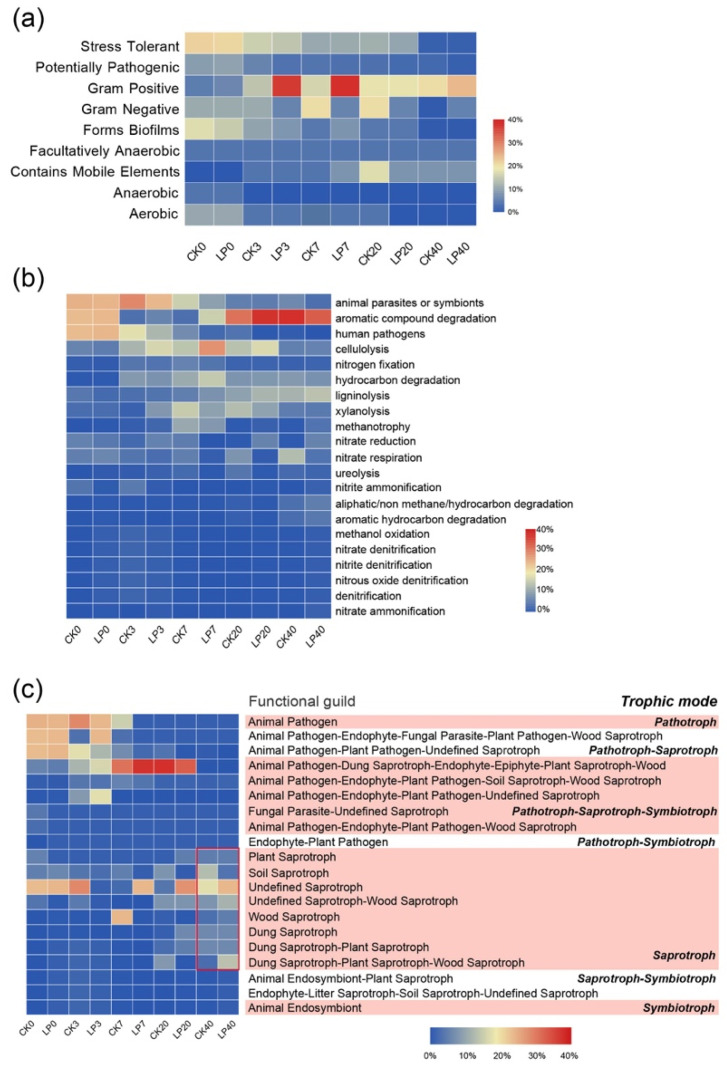
Heatmaps of the dynamics of microbial function in the compost with and without inoculation. (**a**) The bacterial phenotypes based on BugBase. (**b**) Bacterial function prediction based on FAPROTAX. (**c**) The fungal trophic mode and functional guild based on FUNGuild. Red line were the Guilds in final composts.

The bacterial phenotypic characteristics in compost were determined based on the BugBase database to predict and determine bacterial function. Nine potential phenotypes were found in the composting, which included pathogenic, aerobic, anaerobic, facultatively anaerobic, contained mobile elements, biofilm-forming, Gram-negative, Gram-positive and oxidative-stress-tolerant (Figure 4a). During aerobic composting, the RAs of bacterial communities that were aerobic, anaerobic, potentially pathogenic and formed biofilms decreased, but Gram-negative communities containing mobile elements increased. Interestingly, compared with the CK group, the addition of LFPO increased the RAs of Gram-negative bacteria (*p* < 0.05), and fewer potentially pathogenic (average RA of 0.28%) and biofilm-forming bacteria (RA of 1.2%) were listed in LFPO aerobic composting. Bacteria that can form biofilms usually grow and spread by attaching to a fixed material by forming a biofilm pattern and have the characteristics of high temperature resistance and antibiotic resistance, which will increase their potential risk of becoming pathogenic bacteria, resulting in a series of health problems [33,34].

As shown in Figure 4b, FAPROTAX is an artificially constructed database that maps prokaryotic taxa (such as genera) to metabolic or other ecologically related functions [35]. The RA of bacteria involved in hydrocarbon degradation increased, while the RA of animal parasites or symbionts and human pathogens decreased, especially in the LFPO group, and the RA of human pathogens decreased to approximately 0.01% after 7 days of composting. Notably, the RA of bacteria involved in cellulolysis increased rapidly after day 3 (*p* < 0.05), and the RA of bacteria involved in ligninolysis increased in the late composting stages. These trends were more obvious in LFPO compared with CK. The result confirmed the previous results that the thermophilic stage was the main degradation period of cellulose and hemicellulose, and lignin degradation was roughly concentrated in the late stages of composting (Section 3.2). Additionally, LFPO incubation accelerated the degradation of lignocellulose and OM. Similar studies have shown that other *Lysinibacillus* species can also be used as lignocellulosic bacteria, and some have resistance to high temperatures [18,36]. These results can be explained by the addition of microorganisms (LF-N1 and DH-1), which are closely related to the changes in the bacterial phenotype and metabolic function.

#### 3.5.2. Fungal Function Prediction

Figure 4c shows trophic type prediction for fungal communities in samples based on FUNGuild. The fungal trophic modes and guilds showed significant variation during aerobic composting. The fungal trophic modes and guilds were mainly classified into the following parts: pathotrophs (animal pathogens, 23.2%), pathotrophs–saprotrophs (animal pathogens–endophytes–plant pathogens–wood saprotrophs, 24.7%), saprotrophs-symbiotrophs (endophytes–plant pathogens, 13.2%) and saprotrophs (undefined saprotrophs, 8.3%), which existed in the original compost. During composting, saprotrophic fungi increased more in the LFPO group than in the CK group, and saprotrophs (undefined saprotrophs, plant saprotrophs, wood saprotrophs, 63.2%) became the dominant trophic mode in the LFPO group at the end of composting. The RAs of pathotroph-related modes in both groups decreased during composting, and they decreased to less than 0.01% after the 7th day. Pathotrophs–saprotrophs–symbiotrophs (animal pathogens–endophytes–plant pathogens–wood saprotrophs, 12.6%) and saprotrophs (animal pathogens–endophytes–plant pathogens–wood saprotrophs, 13.5%) were dominant in the CK group.

### 3.6. Co-Networks in Microbial Communities

**Figure 5:**
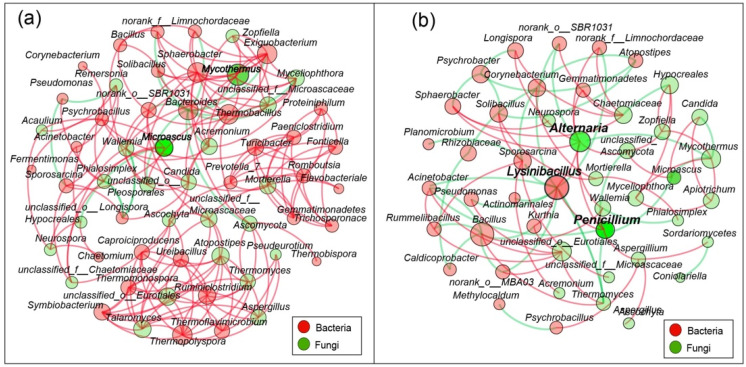
Network analysis showing the cooccurrence based on the interaction relationship among dominant genera. The control group (**a**); LF-N1 and DH-1 inoculation groups (**b**). Red edges: positive correlation; green edges: negative correlation. The size of the nodes shows the average RA of the taxa.

In Figure 5, for a clearer interpretation of the effects of LF-N1 and DH-1 on the microbial communities, cooccurrence networks of the CK group and LFPO group are presented based on the relationships between bacterial and fungal genera, as shown in Figure 5. The figure shows that the LFPO inoculation group decreased the graphic density of the correlation, indicating that the cooccurrence network was simplified with LFPO inoculation compared with the CK, especially for the topological properties of the network, which showed the average weighted degree (5.934 in the CK group and 3.447 in the LFPO group, Table S1). Furthermore, the cooccurrence network among bacterial and fungal genera presented more negative correlations and fewer positive correlations with each other in the LFPO group during composting compared to the CK group. The microorganisms with the highest degree in the LFPO group were mainly *Lysinibacillus* (a degree of 12), followed by *Alternaria* (a degree of 10) and unclassified_o_Eurotiales (a degree of 10). Interestingly, in the LFPO group, *Lysinibacillus*, which was previously confirmed as the most abundant taxa (RA 25.8%), was a key taxon that interacted with other genera since it had negative correlations with six bacterial genera, such as *Acinetobacter*, norank_o_SBR1031, *Sporosarcina* and *Pseudomonas*. The RAs of *Acinetobacter*, *norank_o_SBR1031* and *Sporosarcina* were lower in the LFPO group, but these were the dominant genera in the CK group. These results indicated that *Lysinibacillus* maintained a competitive and inhibitory relationship with other microorganisms and eventually became the dominant flora in the LFPO group in the process of community evolution. In addition, *Lysinibacillus* had positive correlations with three fungal genera, namely *Alternaria*, *Penicillium* and unclassified_o_Eurotiales, which demonstrated that *Lysinibacillus* likely produced various enzymes or certain secondary metabolites, which promoted fungal growth and metabolism. *Penicillium* presented a positive correlation with *Alternaria*, which was confirmed to be the most abundant genus in the LFPO group, while it exhibited negative correlations with *Microascus*, *Acinetobacter* and *Thermomyces*, which were abundant in CK.

### 3.7. Relationships of the Microbial Structure with Physicochemical Properties

**Figure 6:**
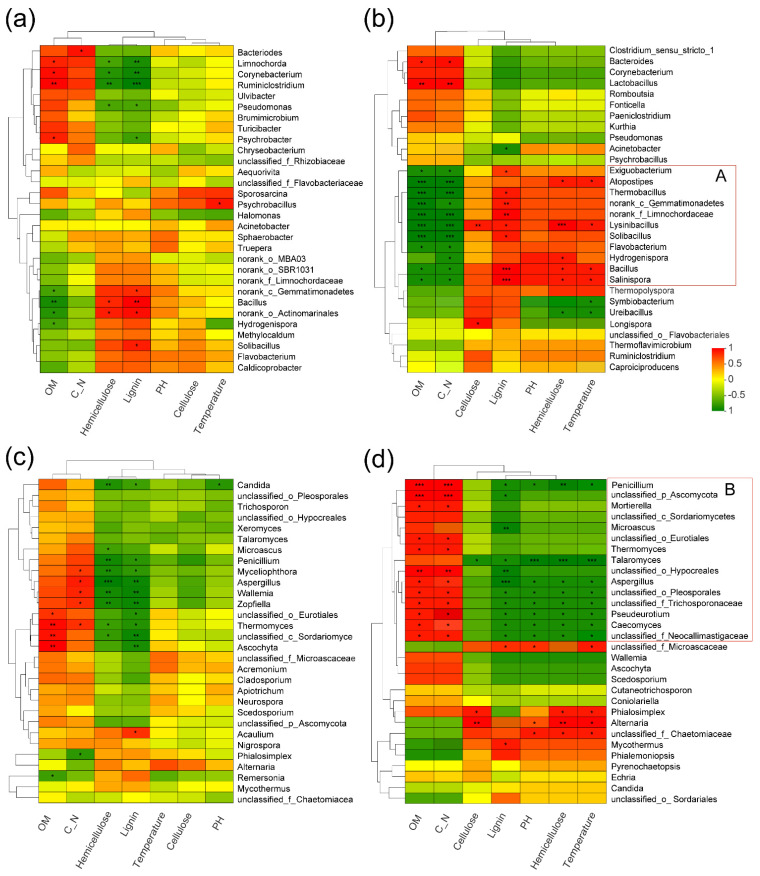
Heatmap showing relationship analysis of physicochemical parameters and the (**a**) bacterial genera in CK, (**b**) bacterial genera in LFPO, (**c**) fungal genera in CK and (**d**) fungal genera in LFPO. (* 0.01 < *p* ≤ 0.05, ** 0.001 < *p* ≤ 0.01, *** *p* ≤ 0.001). Red lines were the microbial cluster.

In Figure 6, the heatmaps show the correlation between microorganisms and alterations in the physicochemical properties during the composting process with and without inoculation. In LFPO, cluster A was represented by *Lysinibacillus* in the bacterial community (Figure 6b), which had a positive correlation with temperature and lignocellulose degradation and a negative correlation with OM degradation and the carbon:nitrogen ratio. Significant positive correlations with temperature were mainly found for *Lysinibacillus*, *Bacillus* and *Salinispora*, and negative correlations were mainly found for *Symbiobacterium* and *Ureibacillus*. However, there were relatively few temperature-related genera in the CK group (Figure 6a). Specifically, *Lysinibacillus* had a significant positive association with temperature and cellulose/hemicellulose degradation (*p* < 0.05), but there was a significant negative association between C/N ratio, the degradation of OM and *Lysinibacillus* (*p* < 0.05). Among the bacterial clusters, *Solibacillus*, *Hydrogenispora*, *Bacillus*, *Salinispora* and *Thermobacillus* were negatively correlated with OM degradation and the C/N ratio (*p* < 0.05) but were negatively related to lignin degradation. The significant positive correlation observed for lignin degradation may be due to the rapid degradation of lignin caused by the general aggregation of lignin-degrading bacteria after the temperature decreased slightly in the later stage of composting. A significant positive association was observed between *Longispora* and cellulose degradation (*p* < 0.05). *Bacteroides* and *Lactobacillus* had a positive relationship with OM degradation and the C/N ratio (*p* < 0.05).

Fungal cluster B, represented by *Penicillium* in the LFPO group, was inversely proportional to temperature, pH and lignocellulose degradation and was positively proportional to OM degradation and the C/N ratio (Figure 6d). Among the organisms, *Talaromyces*, *Aspergillus*, Polyspora unclassified_Pleosporales, unclassified_f_Trichosporonaceae, *Pseudeurotium*, *Caecomyces*, unclassified_f_Neocallimastigaceae, *Penicillium* and other fungi had a significant negative correlation with temperature and lignocellulose degradation (*p* < 0.05). These fungi also had a significantly positive relationship with OM degradation and the C/N ratio (*p* < 0.05). *Penicillium* strain incubation may have affected the fungal community patterns, leading to similar correlations between fungal clusters and environmental factors. In addition, compared with the control group, *Penicillium* improved the correlations between the fungal community with OM degradation and the C/N ratio in the compost (Figure 6c), and the group to which the strains were added had significantly more temperature-related genera, such as *Alternaria*, *Phialosimplex*, unclassified_f_Chaetomiaceae and unclassified_f_Microascaceae (*p* < 0.05). The dominant fungi in LFPO, *Alternaria* and *Phialosimplex* were also positively associated with cellulose and hemicellulose decomposition (*p* < 0.01). Although the control group had relatively few fungal genera significantly related to temperature compared with the LFPO group, similar to the LFPO group, *Penicillium* and *Aspergillus* in the CK group were significantly negatively related to the degradation of lignocellulose (*p* < 0.05).

## 4. Conclusions

In this study, two functional strains, *Lysinibacillus* sp. LF-N1 and *Penicillium oxalicum* DH-1, were mixed and inoculated into cow manure and wheat straw compost (LFPO) to explore their effects on composting performance and microbial community dynamics. Compared with the CK, the microbial inoculants raised the maximum composting temperature to 62.8 °C, the GI value increased by 30%, and the lignocellulose degradation rate increased by more than 13%. Microbial diversity data indicated that the microbial structures were significantly changed after inoculation, and the numbers of LF-N1 and DH-1 were obviously higher compared to those in the CK group. Through cooccurrence network analysis, we found that a large number of microorganisms were significantly associated with the inoculants, and the interactions in the microbial community were clarified. In summary, the two strains, LF-N1 and DH-1, are indeed effective in promoting the composting process, which will provide the basis for the functional application of the strains in the future. We will continue to screen and compound functional strains to identify the most effective strains and then obtain a more effective inoculant combination.

## Figures and Tables

**Table 1:** Chemical constituents of the agricultural wastes.

Materials	Cow Manure	Wheat Straw
Moisture (%)	61.6 ± 1.9	8.7 ± 1.7
pH	7.92 ± 0.07	6.63 ± 0.05
OM (Organic matter, g·kg^−1^))	785.4 ± 2.3	653 ± 2.3
TOC (Total organic carbon, g·kg^−1^)	312 ± 1.5	443 ± 2.3
TKN (Total Kjeldahl nitrogen, g·kg^−1^)	14.7 ± 0.02	7.9 ± 0.01
C/N (Carbon: nitrogen ratio)	29.1 ± 0.3	24.2 ± 0.5
Cellulose (%)	33.9 ± 1.3	39.4 ± 1.8
Hemicellulose (%)	26.5 ± 1.6	28.4 ± 1.2
Lignin (%)	13.5 ± 0.2	4.6 ± 0.03

The values are shown as the mean ± standard deviation of three replicates.

**Table 2:** Variations in physico-chemical properties during composting.

Items	Time (Day)					SEM	Significance		
	0	3	7	20	40		TR	TI	TR × TI
pH									
CK	7.67 ± 0.01 Ea	8.36 ± 0.01 Aa	8.21 ± 0.01 Ba	7.89 ± 0.01 Db	8.04 ± 0.06 Ca	0.004	*	***	***
LFPO	7.68 ± 0.02 Ea	8.31 ± 0.03 Aa	8.18 ± 0.01 Ba	8.03 ± 0.01 Ca	7.87 ± 0.01 Db				
TOC (g·kg^−1^)									
CK	374.6 ± 10.59 Aa	349.3 ± 4.16 ABa	336.0 ± 6.25 ABa	286.1 ± 58.0 Ba	282.3 ± 10.4 Ba	3.675	0.134	***	0.673
LFPO	373.1 ± 13.1 Aa	343.6 ± 9.60 Ba	321.7 ± 4.04 Ba	282.7 ± 9.61 Ca	269.6 ± 5.58 Cb				
TN (g·kg^−1^)									
CK	14.6 ± 0.51 Aa	13.9 ± 0.21 Aa	15.4 ± 0.34 Aa	14.6 ± 2.86 Aa	16.1 ± 0.29 Aa	0.182	0.331	*	0.759
LFPO	14.5 ± 0.72 BCa	14.3 ± 0.66 Ca	16.3 ± 0.44 Aa	15.6 ± 0.34 ABCa	16.8 ± 0.49 ABa				
C/N									
CK	25.6 ± 0.25 Aa	24.9 ± 0.11 A^a^	21.7 ± 0.32 Ba	19.5 ± 0.17 Ca	18.7 ± 0.36 Da	0.075	***	***	*
LFPO	25.5 ± 0.41 Aa	23.9 ± 0.61 B^b^	19.7 ± 0.35 Cb	18.1 ± 0.26 Db	16.4 ± 0.78 Eb				
GI									
CK	32.3 ± 1.15 Ea	44.7 ± 0.45 Db	73.5 ± 0.56 Cb	101.5 ± 2.76 Bb	125.4 ± 5.50 Ab	0.773	0.331	***	***
LFPO	33.1 ± 1.05 a	55.8 ± 0.95 a	88.2 ± 1.05 a	136.0 ± 6.45 a	177.1 ± 9.72 a				

CK: the control group; LFPO: the inoculated group. Capital letters indicate a significant difference between different composting times (*p* < 0.05). Lowercase letters indicate a significant difference between CK and LFPO (*p* < 0.05). The values are shown as the mean ± standard deviation of three replicates. SEM = standard error of the means. TR: treatment; TI: time; TR × TI: the interaction between treatment and time; *: *p* < 0.05; ***: *p* < 0.001.

**Table 3:** Variations in the physical and chemical parameters.

Items	Time (Day)					SEM	Significance		
	0	3	7	20	40		TR	TI	TR × TI
OM (organic matter, g·kg^−1^)									
CK	782.3 ± 17.6 Aa	736.1 ± 6.0 Ba	679.2 ± 4.6 Ca	543.4 ± 10.5 Da	521.6 ± 3.0 Da	0.225	*	***	***
LFPO	778.7 ± 17.4 Aa	665.7 ± 10.7 Bb	527.3 ± 15.5 Cb	460.9 ± 16.1 Db	420.1 ± 10.5 Eb				
Cellulose (g·kg^−1^)									
CK	363.3 ± 15.5 Aa	353.6 ± 13.2 ABa	319.6 ± 14.1 Ba	244.3 ± 10.4 Ca	193.6 ± 11.0 Da	2.203	***	***	***
LFPO	364.0 ± 8.54 Aa	321.3 ± 14.0 Bb	235.0 ± 11.5 Cb	154.3 ± 13.0 Db	125.0 ± 6.0 Db				
Hemicellulose (g·kg^−1^)									
CK	275.6 ± 17.6 Aa	232.3 ± 8.50 Ba	172.5 ± 10.0 Ca	123.7 ± 10.2 Da	93.6 ± 10.5 Da	1.927	***	***	**
LFPO	278.3 ± 16.5 Aa	192.3 ± 11.0 Bb	124.7 ± 3.51 Cb	71.6 ± 2.31 Db	53.8 ± 3.02 Db				
Lignin (g·kg^−1^)									
CK	104.6 ± 2.51 Aa	98.0 ± 6.00 Aa	89.6 ± 2.08 Ba	69.0 ± 3.00 Ca	51.0 ± 8.54 Da	1.018	*	***	0.787
LFPO	104.0 ± 2.65 Aa	89.3 ± 7.64 ABa	84.3 ± 10.1 Ba	64.6 ± 2.51 Ca	44.3 ± 3.21 Da				

CK: the control group; LFPO: the inoculated group. Capital letters indicate a significant difference between different composting times (*p* < 0.05). Lowercase letters indicate a significant difference between the CK and LFPO (*p* < 0.05). The values are shown as the mean ± standard deviation of three replicates. SEM = standard error of means. TR: treatment; TI: time; TR × TI: the interaction between treatment and temperature; *: *p* < 0.05; **: *p* < 0.01; ***: *p* < 0.001.

**Table 4:** Alpha diversity of the microbial structures in compost.

Community	Treatment	Time (Day)	Coverage	Chao 1	Ace	Shannon	Simpson
				Richness		Diversity	
Bacterial community	CK	0	1.0	783.29 ± 3.8	892.26 ± 41.8	3.20 ± 0.01	0.28 ± 0.01
		3	1.0	620.12 ± 21.3	716.45 ± 31.81	1.54 ± 0.02	0.33 ± 0.01
		7	1.0	404.09 ± 31.1	389.50 ± 21.3	2.13 ± 0.06	0.30 ± 0.01
		20	1.0	476.01 ± 12.7	485.06 ± 24.7	2.96 ± 0.07	0.18 ± 0.01
		40	1.0	597.19 ± 14.3	659.16 ± 41.2	2.89 ± 0.21	0.12 ± 0.08
	LFPO	0	1.0	749.29 ± 4.26	878.35 ± 34.6	3.30 ± 0.01	0.25 ± 0.12
		3	1.0	647.17 ± 6.89	759.72 ± 12.9	1.51 ± 0.07	0.43 ± 0.17
		7	1.0	456.72 ± 9.12	518.17 ± 14.7	1.37 ± 0.05	0.41 ± 0.05
		20	1.0	596.57 ± 13.2	657.00 ± 31.5	2.38 ± 0.21	0.25 ± 0.01
		40	1.0	652.08 ± 21.1	730.58 ± 23.4	2.33 ± 0.01	0.26 ± 0.01
Fungal community	CK	0	1.0	323.25 ± 11.4	283.07 ± 25.6	4.21 ± 0.22	0.03 ± 0.01
		3	1.0	223.44 ± 21.8	188.42 ± 32.5	3.19 ± 0.09	0.17 ± 0.01
		7	1.0	123.74 ± 22.1	172.69 ± 26.5	3.88 ± 0.03	0.14 ± 0.02
		20	1.0	197.89 ± 11.6	192.20 ± 24.3	3.01 ± 0.02	0.08 ± 0.03
		40	1.0	200.75 ± 27.8	219.31 ± 12.5	3.13 ± 0.07	0.11 ± 0.01
	LFPO	0	1.0	303.05 ± 17.6	246.86 ± 21.6	4.40 ± 0.04	0.11 ± 0.01
		3	1.0	293.73 ± 24.3	221.06 ± 14.3	3.87 ± 0.14	0.20 ± 0.07
		7	1.0	173.67 ± 3.87	185.17 ± 24.3	2.88 ± 0.03	0.12 ± 0.01
		20	1.0	227.89 ± 9.12	203.87 ± 14.5	3.41 ± 0.02	0.18 ± 0.01
		40	1.0	226.25 ± 21.8	226.99 ± 25.9	3.85 ± 0.03	0.21 ± 0.01

LFPO: inoculation group; CK: control group. The number following LFPO/CK indicates the sampling time (day). The mean ± standard deviation of three replicates. Red line were the Guilds in final composts.

## Data Availability

The datasets generated for this study can be found in Sequence Read Archive under BioProject, BioProject ID: PRJNA694866 (https://www.ncbi.nlm.nih.gov/ accessed on 16 March 2022).

## Supplementary Materials

The following supporting information can be downloaded at: https://www.mdpi.com/article/10.3390/microorganisms10040709/s1, Figure S1: Composting reactor. Figure S2: The relative abundance of microbial taxa at the phylum level; Figure S3: The relative abundance of microbial taxa at the species level; Table S1: The topological parameters of the co-networks.
