# Supplementary material for: Dynamic Microstructure Assembly Driven by Lysinibacillus sp. LF-N1 and Penicillium oxalicum DH-1 Inoculants Corresponds to Composting Performance
Source: Microorganisms. 2022 Mar 25;10(4):709. doi: 10.3390/microorganisms10040709 (PMC9028265; doi:10.3390/microorganisms10040709)
Supplement: Supplementary file 1 [file microorganisms-10-00709-s001.zip › microorganisms-1632020-supplementary.pdf]

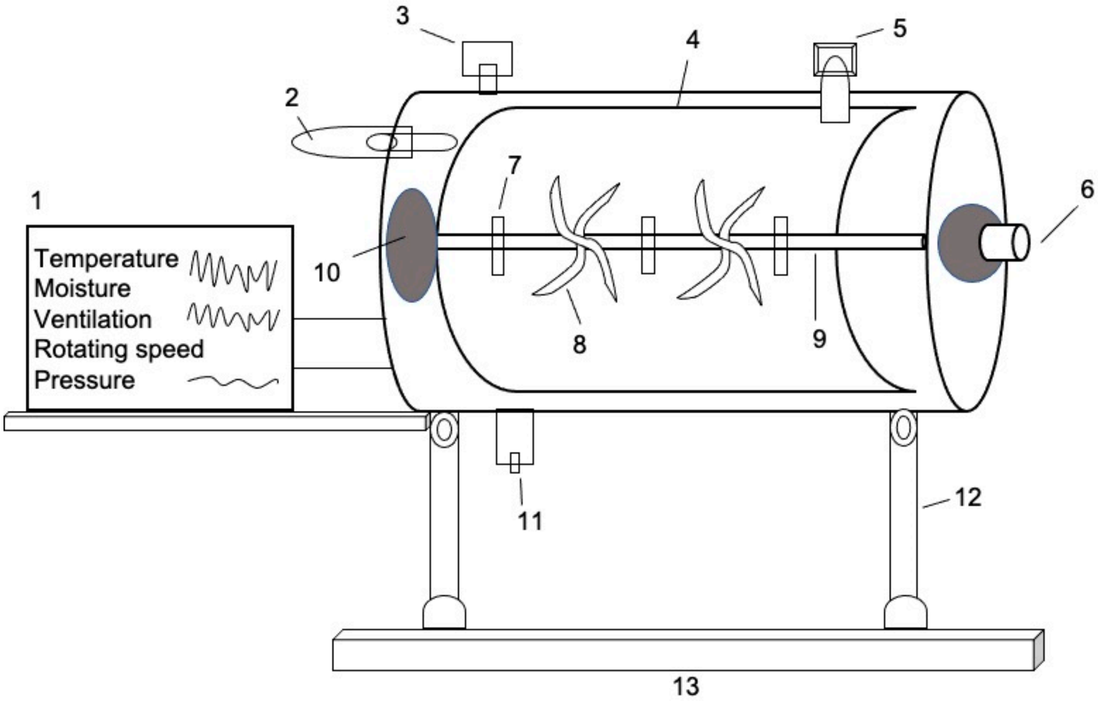

Figure S1. Composting reactor. 1. Screen; 2. air outlet; 3. water intake; 4. reactor; 5. air intake; 6. sampling collection port; 7. temperature sensor; 8. stirring paddle; 9. rotating shaft; 10. raw material inlet; 11. water outlet; 12. fixed rod; 13. support bottom plate.

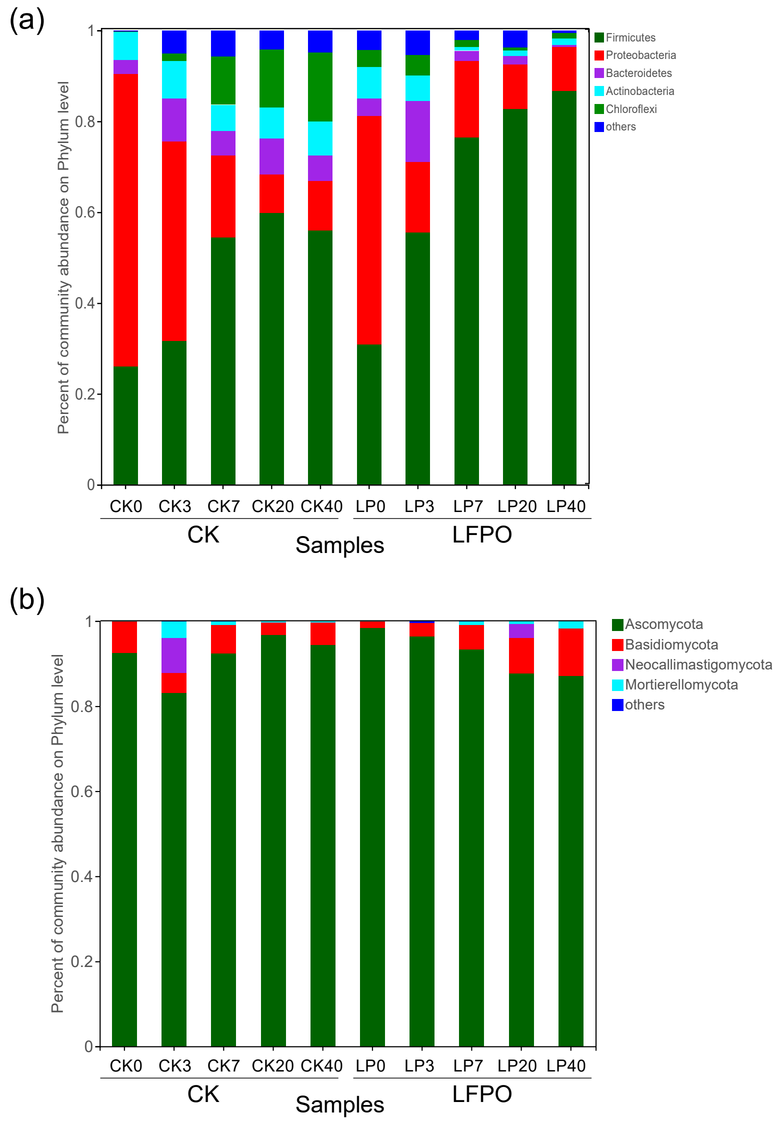

Figure S2. (a) The relative abundance of bacterial taxa at the phylum level. (b) The relative abundance of fungal taxa at the phylum level;

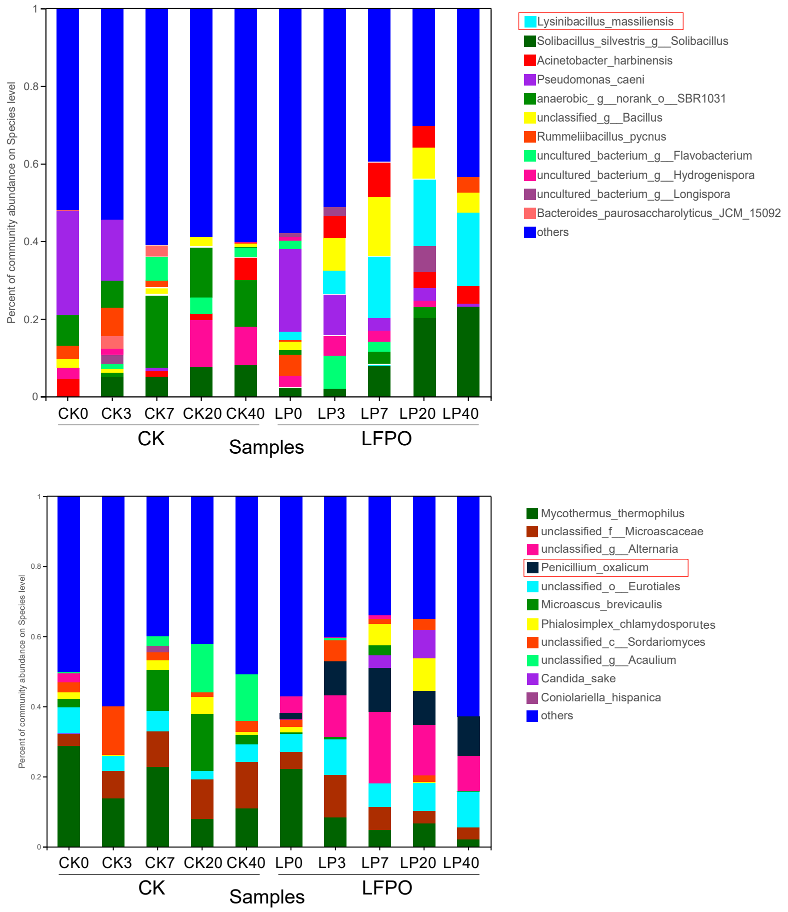

Figure S3. (a) The relative abundance of bacterial taxa at the species level. (b) The relative abundance of fungal taxa at the species level;

Table S1. The topological parameters of co-networks.

Object	Parameters	CK	LFPO
Network Overview	Average Degree	5.934	3.447
	Network Diameter	12	9
	Graph Density	0.099	0.075
	Modularity	0.654	0.641
	Connected components	3	3
Node Overview	Nodes number	61	46
	Clustering Coefficient	0.337	0.632
	Eigenvector Centrality (100run)	0.0097	0.0054
Edge Overview	Edge number	181	95
	Path length	5.058	4.072
